# Influence of slice thickness on MR flow quantification in turbulent jets

**DOI:** 10.1186/1532-429X-13-S1-P363

**Published:** 2011-02-02

**Authors:** Matthias A Dieringer, Andreas Greiser, Jeanette Schulz-Menger

**Affiliations:** 1 Experimental and Clinical Research Center (ECRC), Charite' - University Medicine Campus Berlin Buch, Berlin, Germany; 2 Siemens Healthcare Sector, Erlangen, Germany

## Introduction

Magnetic Resonance (MR) based flow quantification is of high interest in several cardiac diseases, e.g. valvular stenosis. However, accurate results remain challenging in several conditions. Recently, some contributors could be defined including echo time [1] (TE) and background phase errors [2]. We investigated the influence of slice thickness on flow quantification in turbulent flow.

## Methods

**Figure 1:**
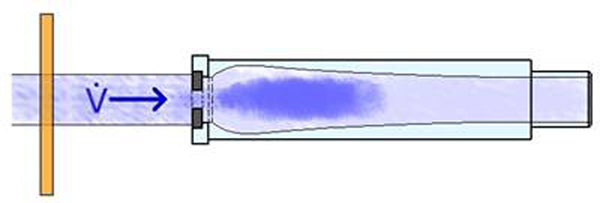
Slice positioning

A flow phantom was developed, which disposes of an adjustable pump and a model similar to the shape of a human aorta (Figure 1). To imitate an aortic stenosis, a Perspex plate with a borehole (D=11.5mm; A≈1cm^2^) was introduced. Retrospectively gated phase contrast cine imaging (in-plane resolution 1.8x1.3mm) was applied to obtain flow data from an Avanto 1.5T MR system (Siemens Healthcare Sector, Erlangen, Germany). Tested sequence settings were *a)* RF pulse length=1000us, maximum flow encoding gradient strength=10mT/m, TE=3.1ms; *b)* RF pulse length=400us, maximum flow encoding gradient strength=20mT/m, TE=2.2ms; *c)* RF pulse length=400us, maximum flow encoding gradient strength=20mT/m, TE=3.1ms. Tested slice thicknesses ranged from 4.5mm to 10mm. Steady but turbulent flow (300ml/s) was measured at 2.5cm behind the stenosis (Figure 1b) with 20 calculated cardiac phases (yielding 20 flow values). Flow quantification was done using CMR42 (Circle CVi, Calgary, Canada). Average flow deviation and its standard deviation were calculated.

## Results

**Figure 2:**
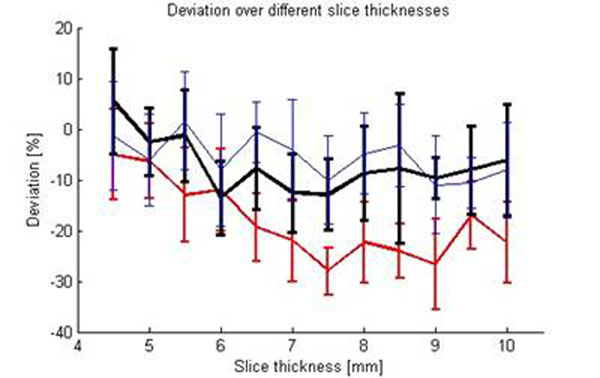
Flow deviation dependent on the slice thickness with *a)* TE=3.1ms (red), *b)* TE=2.2ms (black), *c)* TE=3.1ms (blue). Maximum flow underestimation was found at *a)* (-27%, ST=7.5mm), maximum overestimation was found at *b)* (9%, ST=4.5mm)

Figure 2 shows the measured flow deviations for the tested settings (a-c) over the different slice thicknesses. The maximum flow deviation was measured in case of an RF pulse of 1000us duration and a maximum flow encoding gradient strength of 20mT/m. Increasing TE did not show additional flow errors. Measurements with slice thicknesses below 6mm were found to be higher than with slice thicknesses above.

## Conclusion

Our results show that slice thickness influences flow quantification in the presence of turbulent flow in certain sequence configurations. Decreasing the time of flow encoding by increasing the flow encoding gradient strength seems sufficient to mitigate flow errors for all slice thicknesses. Further investigations have to be conducted to explain the impact of the slice thickness on flow quantification in-vivo.
